# PANDA: Protein function prediction using domain architecture and affinity propagation

**DOI:** 10.1038/s41598-018-21849-1

**Published:** 2018-02-22

**Authors:** Zheng Wang, Chenguang Zhao, Yiheng Wang, Zheng Sun, Nan Wang

**Affiliations:** 1 Department of Computer Science, University of Miami, 1364 Memorial Drive, P.O. Box 248154, Coral Gables, FL 33124 USA; 2 School of Computing, University of Southern Mississippi, 118 College Drive #5106, Hattiesburg, MS 39406 USA; 3 Department of Mathematics and Computer Science, The Citadel, 171 Moulrie Street, Charleston, SC 29409 USA; 4 Department of Computer Science, New Jersey City University, 2039 Kennedy Blvd, Jersey City, NJ 07305 USA

## Abstract

We developed PANDA (Propagation of Affinity and Domain Architecture) to predict protein functions in the format of Gene Ontology (GO) terms. PANDA at first executes profile-profile alignment algorithm to search against PfamA, KOG, COG, and SwissProt databases, and then launches PSI-BLAST against UniProt for homologue search. PANDA integrates a domain architecture inference algorithm based on the Bayesian statistics that calculates the probability of having a GO term. All the candidate GO terms are pooled and filtered based on Z-score. After that, the remaining GO terms are clustered using an affinity propagation algorithm based on the GO directed acyclic graph, followed by a second round of filtering on the clusters of GO terms. We benchmarked the performance of all the baseline predictors PANDA integrates and also for every pooling and filtering step of PANDA. It can be found that PANDA achieves better performances in terms of area under the curve for precision and recall compared to the baseline predictors. PANDA can be accessed from http://dna.cs.miami.edu/PANDA/.

## Introduction

The goal of protein function prediction is to predict the Gene Ontology (GO) terms^1^ for a query protein given its amino acid sequence. Majority of the existent methods make predictions based homologue searching, whereas there are methods making predictions based on protein structure and text mining.

In terms of homologue searching, the theory behind is that if two proteins are significant homologues, they may share similar functions. Although research shows that the sequential identify must reach 80% in order for two proteins to share similar Enzyme Classification (EC)^2^, homologue searching usually still is the starting point of protein function prediction. There are many methods that assign different weights to the GO terms of homologues based on the homologue-searching tools’ scores, for example, the e-value of PSI-BLAST^3^. A more sensitive homologue searching method is profile-profile alignment, which generates and then aligns a profile of the query protein with the profile of each protein in the database^4^. HHsuit (HHblits)^5^ is a tool that implements profile-profile searching and has been used in both protein function prediction^6,7^ and protein structure prediction (for template identification)^8,9^.

Hidden Markov model (HMM) has been used to search for protein domains in a query protein. A domain is a segment of protein that has reserved structural or functional properties. Pfam^10^ is one of the most comprehensive databases for protein domains. Based on all the amino acid sequences of a type of protein domain, an HMM can be built by multiple sequence alignment. An HMM model can be used to identify a specific type of domain from query protein sequence. Many of the existent Pfam domains have GO terms annotated. Therefore, using this known information and domain detection algorithm, such as PfamScan^11^, GO terms can be annotated.

The co-occurrence patterns of a certain protein domain or domain architecture can provide useful information for protein function prediction. Our previous study has used a protein domain co-occurrence network to infer species phylogenic relationship and predict protein functions^6,12^. Moreover, Pfam2GO^13^ is a software package that uses Bayesian statistics to represent the relationship between domain architectures and GO terms. This tool originally uses PfamScan, a profile-sequence alignment tool, to detect protein domains. In our research, we use the more sensitive profile-profile alignment approach to detect domains. Therefore, the Bayesian models have been trained with a larger number of more accurate examples.

Orthologues usually retain the same protein functions in the course of evolution. Therefore, it is also a useful approach to search against orthologues databases and use the annotated orthologous proteins to infer the function of query protein. COGs (for prokaryotes and unicellular eukaryotes)^14^ and KOGs (for eukaryotes)^15^ are two datasets containing orthologue clusters of proteins. Another widely used orthologue database is EggNOG^16^ that has also been used for protein function prediction^4^.

There are in total three categories of GO terms: molecular function, biological process, and cellular component and more than 50,000 GO terms. The GO terms of each category form a directed acyclic graph. The homologue based methods along with other HMM or orthologues based methods usually find a large number of candidate GO terms existent as the nodes in the graph. It is not trivial to infer the real GO terms from sometimes hundreds of candidate GO terms based on the GO term graph topology. Graph-based affinity propagation^17^ or clustering of GO terms is the novel approach PANDA uses to address this challenge.

Our tool PANDA integrates both profile-sequence alignment tools (PSI-BLAST, PfamScan) and profile-profile alignment tool (HHblits) to detect remote homologues by searching against multiple databases including UniProt, Pfam, KOG, COG, and SwissProt. These databases contain information about proteins, protein domains, and orthologues. A Bayesian model, based on tool Pfam2GO, is used to infer candidate GO terms from domain architectures. All of the candidate GO terms found by all approaches are clustered using an affinity propagation algorithm^17^ based on the semantic similarities between GO terms. The final predictions are the centroid nodes (GO terms) of each cluster.

## Results

### Benchmark dataset and strategy

We downloaded the GO databases on March 15^th^ 2014 and May 30^th^ 2015. From the proteins added in the later release, we selected 8,420 proteins having the GO term evidence code EXP, IDA, IMP, IGI, IEP, TAS, and IC. These are experimentally determined GO functions. We eliminated the proteins having the GO term GO:0005515 (*protein binding*) as the leaf node because that GO term is considered not informative^7^. We also eliminated the proteins that only have the three root GO terms in molecular function, biological process, and cellular component.

Because we only let PANDA search against databases (details see Materials and Methods) released at no later than September 2013 for homologue detection, the functions of these 8,420 proteins were not available for PANDA when it made predictions. In this way, our benchmarking is a blind test. We further applied CD-HIT^18^, a sequence clustering tool based on sequence identify, onto the 8,420 proteins with sequence identify cut-off set at 0.7 to eliminate redundancy. CD-HIT generated 6,727 clusters; and we kept only one representative sequence in each cluster that is the centroid of the cluster. From these 6,727 proteins, we randomly selected 1,109 proteins as our benchmarking dataset.

Furthermore, when we use a searching tool such as PSI-BLAST or HHsuit to search against protein sequence databases, we eliminate the hits with e-value equals zero or coverage equals 100%. In this way, we make sure that we are not testing the abilities to retrieve GO terms from a database, instead, the abilities to make predictions on unknown proteins that cannot be found directly from a database.

### Precision and recall

The first benchmarking criterion is based on precision and recall. The precision of a target protein *i* is calculated as

1 $$pri=\frac{{\sum }_{f}I(f\in Pi\wedge f\in Ti)}{{\sum }_{f}I(f\in Pi)}$$

The recall of a target protein *i* is calculated as

2 $$rci=\frac{{\sum }_{f}I(f\in Pi\wedge f\in Ti)}{{\sum }_{f}I(f\in Ti)}$$

where *P*_*i*_ represents the set of predicted GO terms with predicted confidence score >*t*, *T*_*i*_ represents the set of experimentally determined GO terms, and *f* is a GO term.

**Figure 1:**
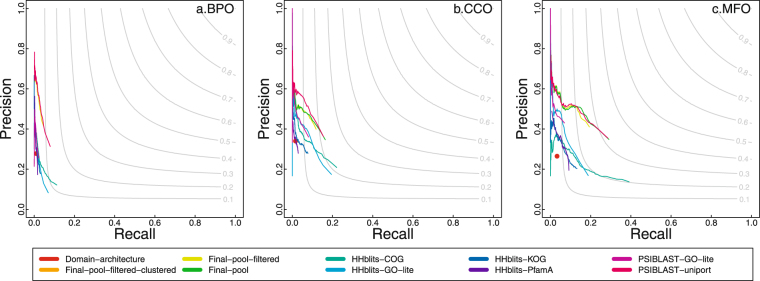
The precision-recall plot of PANDA and other predictors. The predicted and real GO terms are not propagated to the root in the GO directed acyclic graph; and two GO terms are considered a match only if they are the same. The “Final-pool” is based on predictions pooled from all predictors before filtered by Z-score. The “Final-pool-filtered” is before applying GO term clustering using affinity propagation but after filtered by GO term Z-score. The “Final-pool-filtered-clustered” is after the clustering and second round of filtering that is based on clustering results. “Domain-architecture” is a red dot in the plot because we assign a constant confidence score to all predictions from it (see Methods for details).

We perform two versions of evaluations in terms of precision and recall. The first one, as shown in Fig. 1, is a strict benchmarking, in which predicted and real GO terms are not propagated to the root in the GO directed acyclic graph and two GO terms are considered a match only if they are exactly the same.

**Figure 2:**
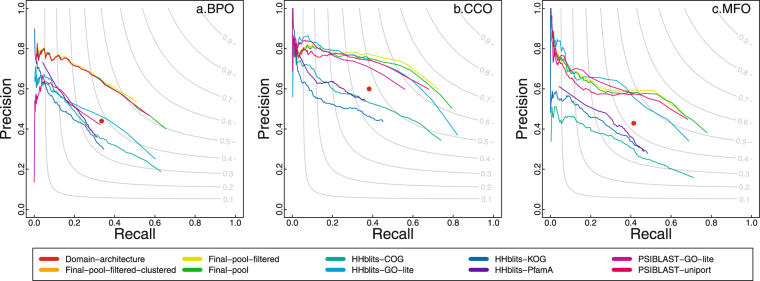
The precision-recall plot of PANDA and other predictors with the lines indicating the values of *area under the curve*. The predicted GO terms and the real GO terms are propagated to the root in the directed acyclic graph. It can be found that by combining the baseline predictors, PANDA’s Final-pool-filtered step achieves the highest area under the curve. Notice that the line of Final-pool-filtered-clustered, which is integrated in PANDA’s server, is overlapping with Final-pool-filtered because the second round of filtering after clustering (Final-pool-filtered-clustered) is not removing more GO terms. Most of the low-quality GO terms have already been filtered out by the first round of filtering (Final-pool-filtered).

In Fig. 2, the predicted GO terms and the real GO terms are propagated to the root in the directed acyclic graph. During the propagation, a GO term’s confidence score equals the maximum confidence score among all of its direct and indirect children GO terms. Figure 2 is not as strict as Fig. 1 because two GO terms may share common ancestor GO terms after being propagated but has been used as the official evaluation approach by CAFA1^7^ and CAFA2^19^.

Among the seven baseline predictors (Domain_architecture, HHblits-COG, HHblits-GO-lite, HHblits-KOG, HHblits-PfamA, PSI-BLAST-GO-lite, and PSI-BLAST-uniprot), the PSI-BLAST searching against UniProtKB (PSI-BLAST-uniprot) performs the best. By combining these baseline predictors and multiple rounds of filterings, PANDA achieves better performance compared to all baseline predictors.

As shown in Figs 1 and 2, we have benchmarked three steps of PANDA: “Final-pool”, which is after directly pooling predictions from seven baseline predictors; “Final-pool-filtered”, which is after adding a Z-score based filtering step; and “Final-pool-filtered-clustered”, which is after adding a clustering step and second round of filtering.

It can be found from Fig. 2 that the pooling process achieves better performances than any baseline predictors. The first round of filtering based on Z-score further improves the performances. However, the clustering and second-round of filtering do not continue improving the performance (the “Final-pool-filtered-clustered” line in Figs 1 and 2 is overlapping with the line of “Final-pool-filtered”).

The reason of this is because PANDA only removes the clusters of GO terms with a Z-score < −2. We have tested using higher Z-score threshold such as −1, 0, and 1. However, a Z-score threshold higher than −2 makes the performance worse. This indicates that the clustering followed by the second round of filtering may not be necessary because the first round of filtering may have already removed all the low-quality GO terms. However, in PANDA server we still keep this step with Z-score threshold set to −2 to ensure extremely low-quality clusters are removed.

**Figure 3:**
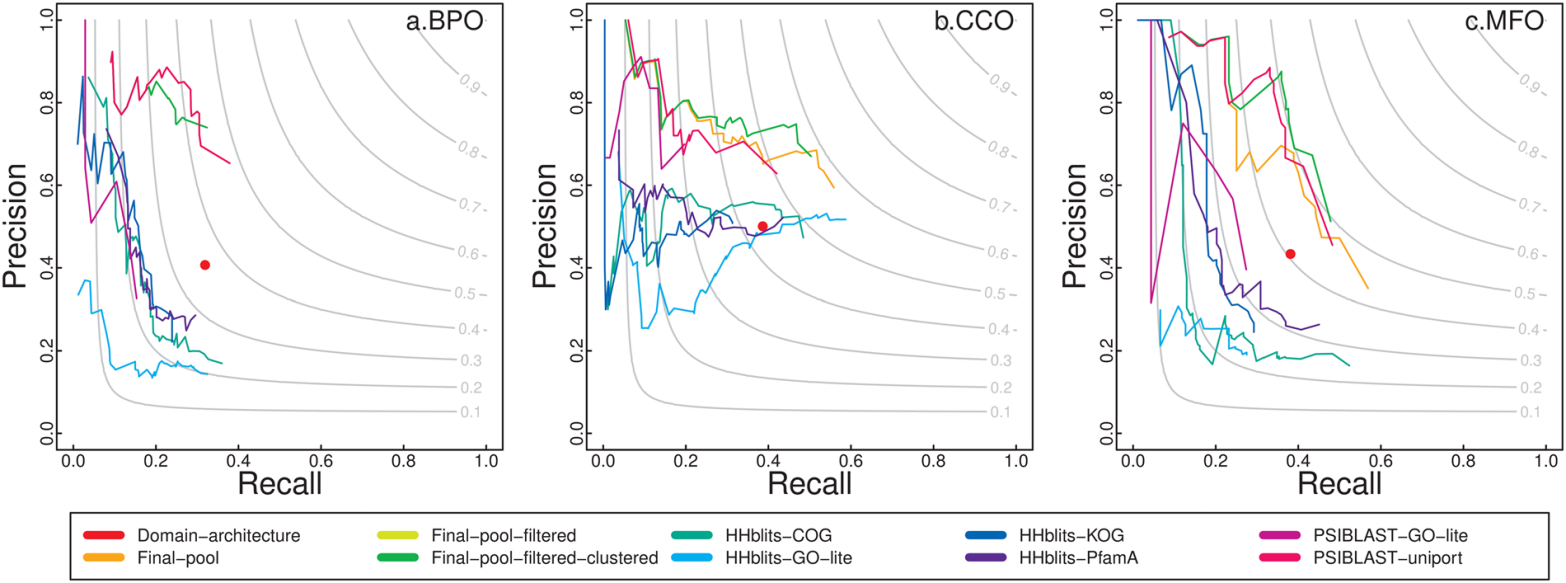
The precision-recall plot of the predictors PANDA integrates on hard targets. Final-pool is overlapped with Final-pool-filtered for BPO.

**Figure 4:**
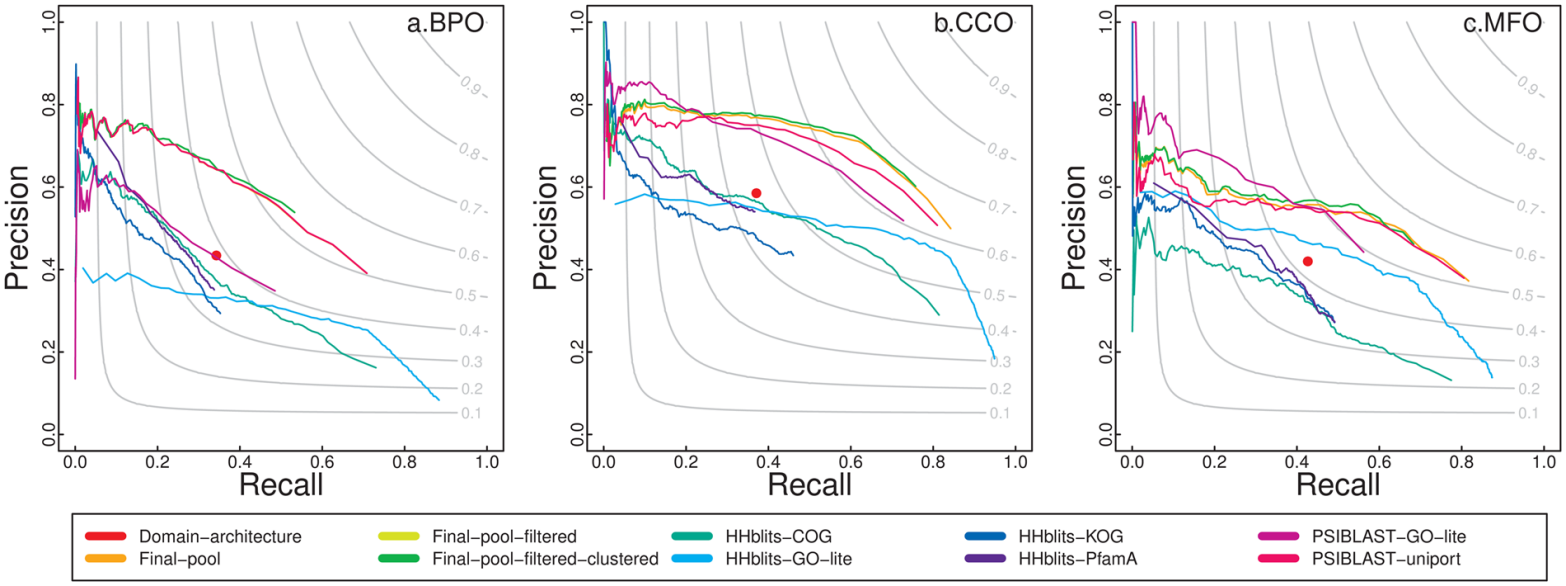
The precision-recall plot of the predictors PANDA integrates on easy targets.

Figure 3 is the precision and recall plot for all the predictors that PANDA integrates on 37 hard targets, on which the highest confidence score gathered from all the predictors is <0.4. Figure 4 is the precision and recall plot on 764 easy targets, on which the highest confidence score is >0.8. Notice that if the searching tool such as PSI-BLAST or HHblits finds a hit with zero e-value and 100% coverage/identity, PANDA removes that hit as it is the query protein itself. From Figs 3 and 4, we can find that our way of integrating PSI-BLAST searching against UniProt performs the best compared to other predictors; and the way PANDA integrates the predictors particularly after the first round of filtering improves the performance for most of the easy and hard cases.

### Semantic similarity between predicted and real GO terms

The previous evaluations based on precision and recall do not consider an important fact about GO terms, which is that two GO terms can have a semantic similarity scoring from 0 to 1. Therefore, although a predicted GO term may be different from the true one, we can still look at how semantically similar they are. Therefore, we have also used the semantic similarity of GO terms to evaluate PANDA and other baseline predictors.

**Figure 5:**
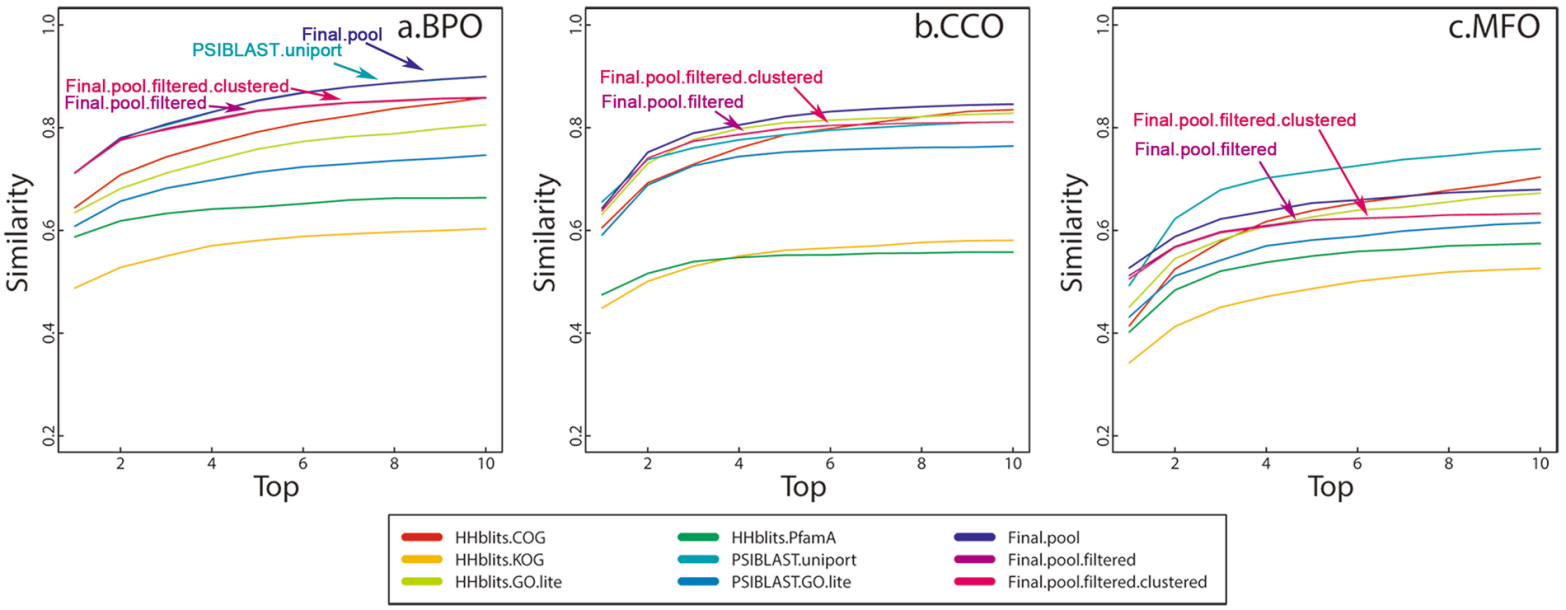
The maximum similarity score between predicted and real GO terms. (**a**) Performances for Biological Process Ontology (BPO). The PSI-BLAST-uniprot and Final-pool are overlapping (the line on the top); whereas Final-pool-filtered-clustered and Final-pool-filtered are overlapping (the second line from the top). (**b**) Performances for Cellular Component Ontology (CCO). Notice that the Final-pool achieves the best performance. Final-pool-filtered-clustered and Final-pool-filtered are overlapping with each other (the third line from the top when considering top 4 predictions). (**c**) Performances for Molecule Function Ontology (MFO), in which the PSI-BLAST-uniprot achieves the highest semantic similarity.

Suppose *T*_*m*_ stands for the *m* true GO terms or experimentally verified GO terms, and *P*_*n*_ the *n* predicted GO terms, the “max similarity” (Fig. 5) by:

3 $$sim\,{\rm{\max }}({T}_{m},{P}_{n})=\mathop{\max }\limits_{1\le i\le m,1\le j\le n}sim(g{o}_{i},g{o}_{j})$$

is the maximum similarity score between two groups (predicted and true) of GO terms. In another word, the “max similarity” is the largest semantic similarity among every pair of GO terms between the predicted group of GO terms and experimentally verified group of GO terms.

Figure 5 is plotted considering top 10 predicted GO terms ranked by confidence score. Not surprisingly, with the increase of number of top predicted GO terms, the max similarity increases. Considering top 3 predictions, PANDA can have a max similarity of 0.8, 0.78, and 0.7 for BPO, CCO, and MFO.

### Evaluations based on *F*_*max*_ score

**Figure 6:**
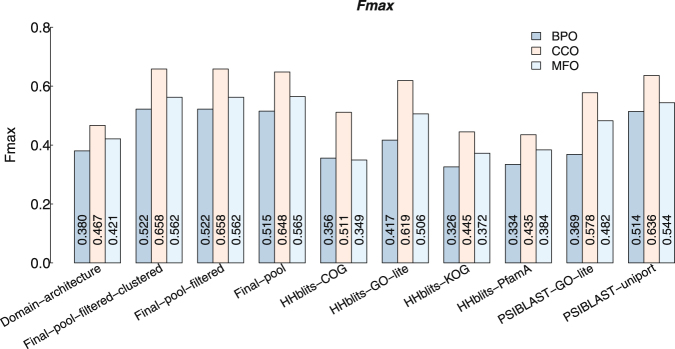
The *F*_*max*_ scores of PANDA and other seven predictors. It can be found that the different steps of filtering and combining baseline predictors of PANDA (starting with Final-) achieve slightly higher performance than PSI-BLAST-uniprot, but significant higher than other baseline predictors.

We use the *F*_*max*_ score, that has been officially used in benchmarking CAFA1 challenge^7^ to benchmark PANDA and other baseline predictors, see Fig. 6. The *F*_*max*_ is a measure to see at which confidence threshold the precision and recall of a predictor are both high in a balanced way. Ideally, when precision and recall both equal 1, the *F*_*max*_ equals 1. However, if precision equals 1 but recall equals 0, the *F*_*max*_ will equal 0, which is the worst scenario. A higher value of *F*_*max*_ indicates better combined performance of a predictor.

For a target protein *i*, the precision and recall for the predicted GO terms with confidence score >= *t* are calculated as Eqs (1) and (2).

The precision and recall for a threshold *t* over all target proteins are calculated as:

4 $$pr(t)=\frac{1}{m(t)}\cdot \sum _{i=1}^{m(t)}pri(t)$$

5 $$rc(t)=\frac{1}{n}\cdot \sum _{i=1}^{n}rci(t)$$

where *m(t)* is the number of target proteins with at least one prediction above threshold *t*, and *n* is the total number of target proteins.

The F_max_ score is then calculated as:

6 $${F}_{{\rm{\max }}}=\mathop{\max }\limits_{t}\{\frac{2\cdot pr(t)\cdot rc(t)}{pr(t)+rc(t)}\}$$

whereas *t*’s range is from 0 to 1 with 0.01 as interval. For domain architecture with no predicted confidence scores, all the GO terms were treated having the same confidence score 0.3. This value is set based on the overall performance of domain architecture. This evaluation also follows the strict criteria, that is, a predicted GO term is considered correct only if it exactly matches a real GO term.

### Evaluations based on ***S***_***min***_ score

The ***S***_***min***_ score is measuring the prediction accuracy considering the specificity of predicted GO terms. GO terms are defined in a directed acyclic graph with the edge meaning “is a” or “part of” relationships between a child node and its parent(s). Therefore, all the descendent nodes are instances of an ancestor node. When making predictions, we would prefer the predictor to make accurate but also specific predictions, i.e., predicting the GO terms with a larger depth value in the directed acyclic graph.

The remaining uncertainty *ru* and the misinformation *mi* are calculated as:

7 $${\rm{ru}}({\rm{t}})=\frac{1}{n}\times \sum _{i=1}^{n}r{u}_{i}(t)$$

8 $${\rm{mi}}({\rm{t}})=\frac{1}{n}\times \sum _{i=1}^{n}m{i}_{i}(t)$$

in which *t* is a confidence threshold varying from 0.01 to 1 with 0.01 as interval; *n* is the number of proteins in the evaluation dataset; and:

9 $$r{u}_{i}(t)=\,\sum _{\,}^{\,}{}_{f}IC(f)\cdot 1{(f\notin {P}_{i}(t){\cap }^{}f\in {T}_{i})}_{}$$

10 $$m{i}_{i}(t)=\,{\sum }^{}\,{}_{f}IC(f)\cdot 1{(f\in {P}_{i}(t){\cap }^{}f\notin {T}_{i})}_{}$$

where *f* represents a GO term; *P*_*i*_*(t)* represents the set of predicted GO term with confidence scores above the threshold *t*; *T*_*i*_ represents the set of real/true GO terms; and *IC* is the information content. The function 1() equals 1 if the condition in the parenthesis is met; otherwise, it equals 0.

In another word, the Eq. (9) is counting the number of GO terms that a predictor has missed to predict, i.e., it is one of the true GO terms of the protein but is not predicted; meanwhile, it also considers the information content of the missed GO term, whereas Eq. (10) is counting the GO terms a predictor predicts but are not true GO terms also with consideration of the information content of the mistakenly predicted GO term(s).

The information content of a GO term $f$ is an indicator of its specificity, which is calculated as:

11 $$I{C}_{f}=-{\mathrm{log}}_{10}\frac{Occu{r}_{f}}{Nu{m}_{all\_terms}}$$

in which $Occu{r}_{f}$ is the number of occurrence for GO term $f$ and all of its descendent GO terms in UniProt database (every GO term is propagated to the root node when calculating number of occurrence); and $Nu{m}_{all\_terms}$ is the total number of occurrences of all GO terms in UniProt database. A larger information content indicates a GO term is less frequently occurred, which indicates it is specific. If information content equals 0, it means the GO term occurs in every protein in the UniProt database, which likely indicates this GO term is the root of the directed acyclic graph, which is not specific at all.

The *S*_*min*_ score^19,20^, or minimum semantic distance, is calculated as:

12 $${S}_{min}={}_{t\,}{}^{min}\,\{\,\sqrt{ru{(t)}^{2}+mi{(t)}^{2}}\}$$

**Figure 7:**
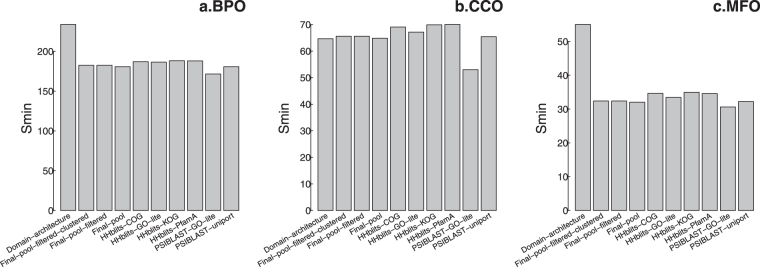
The *S*_*min*_ scores for PANDA and other predictors. It can be found that PSI-BLAST-GO-lite has the lowest *S*_*min*_ scores for CCO. Generally speaking, the other predictors including PANDA are having similar *S*_*min*_ scores.

A larger ${S}_{min}$ score would indicate a predictor has wrongly predicted or missed some GO terms that are specific, which is definitely not what we would like to see for an ideal predictor. Figure 7 shows the ${S}_{min}$ scores for PANDA and other predictors.

### An example illustrating the first filtering of PANDA can remove low-confidence wrong predictions

**Figure 8:**
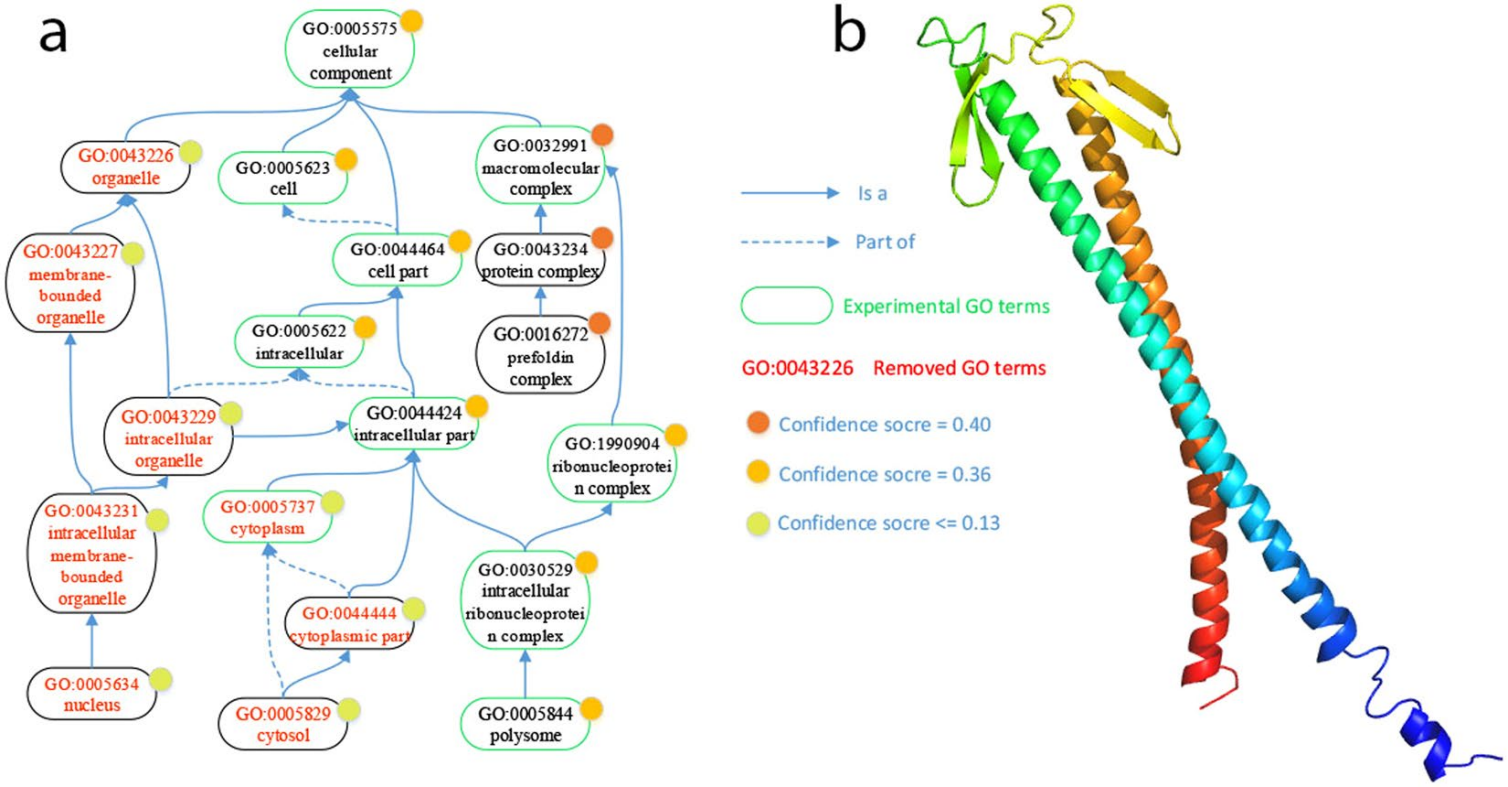
An example showing the first filtering of PANDA (“Final-pool-filtered”) successfully removes some low confidence wrong GO terms from pooled GO terms (“Final-pool”). (**a**) The GO directed acyclic graph showing the GO terms pooled from baseline predictors (all nodes), the GO terms with experimental evidence or the true GO terms (circled by green box), and the GO terms removed by the filtering process (in red color). This example shows that PANDA’s first Z-score based filtering successfully removes the wrongly predicted GO terms. (**b**) The predicted protein structure of the same protein.

To demonstrate that PANDA’s first Z-score-based filtering process can remove low-confidence mistake predictions, we plot Fig. 8(a) (query protein SGD-S344018 from the yeast database SGD^21^), in which the predictions pooled from all the baseline predictors (“Final-pool”) and the GO terms removed are highlighted. The confidence scores of all the predicted GO terms are also illustrated. It can be found that the Z-score filtering step of PANDA removes GO:0044444 (cytoplasmic part) and GO:0005829 (cytosol), but keeps the parent node GO:0005737 (cytoplasm), which is a true GO term of that protein. More interestingly, PANDA removes GO:0005634 (nucleus), which makes all of its ancestor nodes GO:0043231 (intracellular membrane-bounded), GO:0043229 (intracellular organelle), GO:0043227 (membrane-bounded organelle), and GO:0043226 (organelle) removed. These are low-confidence predictions that are not included in the true GO terms set. Figure 8(b) illustrates the predicted 3D structure (by I-TASSER^9^) of the same protein.

It is true that there is a possibility that the removed nodes actually are the functions of that protein, but because of the limitation of current annotations, these GO terms have not been associated with the protein. The ideal solution would be conducting a biological experiment to rule out these GO terms. However, Fig. 2 is plotted on a larger scale, in which it can be seen that the filtering step obviously improves the pooled predictions.

## Discussion

The evaluations demonstrate the performances of PANDA and baseline predictors that PANDA integrates. We have tested three versions of PANDA with different number of steps applied (“Final-pool”, “Final-pool-filtered”, and “Final-pool-filtered-clustered”) in order to find out the contribution of each step. It can be found that our way of integrating the seven predictors can make the pooled-predictions (“Final-pool”) performs better than any of the baseline predictors in terms of the area under the curve in the precision and recall plot.

We find that the way of integrating the baseline predictors is important. The approach applied in PANDA is to assign a weight to each baseline predictor and then only keep top 1 to 5 predictions from a predictor except PSI-BLAST searching against UniProtKB (all kept) because it achieves the best performance among all the six baseline predictors. The intuition is that since PSI-BLAST searching against UniProtKB has achieved the best performance among the six predictors, PANDA should mostly trust its predictions. It is true that HHblits is a profile-profile alignment tool which is more sensitive than PSI-BLAST, a profile-sequence alignment tool, when used to search for remote homologues. However, based on our tests, PSI-BLAST performs better in both hard and easy cases. This may be because that a high sequential similarity does not guarantee functional similarity. It has been found by other research that proteins with highly similar sequences may not share similar functions; and for proteins that share similar functions, they may not share similar sequences. Also, it may be caused by the fact that HHblits can find more hits than PSI-BLAST, which may introduce more noises or false positive GO terms using our way of integrating HHblits’ results. We also find that directly integrating all the predictions from all baseline predictors do not improve performances but make it worse (data not shown). This may be because that it makes lots of low-confidence GO terms or noises integrated.

We also find that for PSI-BLAST searching against UniProtKB, the way of generating confidence scores based on e-value and coverage is important. We have tested other ways of generating confidence scores (data not shown). However, their performances are not comparable to the formula we have used (details see Materials and Methods).

Another contribution this research has made is that we benchmarked the usefulness of multiple filtering approaches. We applied a Z-score based filtering right after pooling all the GO terms. After that, we applied clustering followed by another round of filtering. We find that the first Z-score based filtering right after pooling improves the performance, see Fig. 2, in which the filtered predictions obviously have a better performance than unfiltered. This shows the importance of filtering. However, we found that the filtering after clustering (“Final-pool-filtered-clustered”) is hard to improve the performance anymore. This may be because the Z-score based filtering has already removed noises, so the second round of filtering is not helping that much. However, the second round of filtering can still act as a backup step to remove any extremely low confidence clusters. PANDA server uses the “Final-pool-filtered-clustered” predictions.

Although this research benchmarks multiple predictors and different approaches of filtering the predicted GO terms, it does not apply more advanced techniques such as machine learning. We believe this research has shown that directly combining predictors and applying filtering can only achieve limited improvement from the baseline predictors. In the future, we plan to apply more advanced machine learning approach.

## Conclusions

We have developed PANDA, a protein function prediction server, that performs better than other seven base-line methods in terms of the area under the curve. We find that integrating multiple tools can improve the final performance. However, it is important to assign appropriate weights and only use top predictions. Filtering of pooled GO terms based on Z-score improves the final performance.

## Materials and Methods

The proteins in Gene Ontology Lite (version of Sep 10, 2016) were filtered to only keep the proteins with experimental determined GO terms with the evidence code EXP, IDA, TAS, IMP, IGI, IEP, and IC. There are in total 91,585 proteins left after the filtering. Each query sequence was searched against these protein sequences using PSI-BLAST^22^ with maximum iteration set to 5 and cut-off e-value to 0.01. Each hit (and all the GO terms of the hit) has a score calculated as:

13 $$Score=\frac{-{\mathrm{log}}_{10}e}{40}$$

in which the ${\rm{e}}$ is the e-value and the $Score$ will be cut to a maximum value 1. A specific confidence score for this search is then calculated as:

14 $$Con{f}_{i}^{PSI-BLAST\_GO}=Avg\_Scor{e}_{i}\frac{Occu{r}_{i}}{HitNum}$$

in which $i$ is a specific GO term, $Avg\_Scor{e}_{i}$ is the average score of GO term $i$, $Occu{r}_{i}$ is the total number of occurrence of $i$, and $HitNum$ is the total number of hits. Similarly, PSI-BLAST was used to search against all UniProtKB proteins (551, 193 proteins) and a confidence was calculated in a similar way.

We also applied HHblits^23^ to search against the filtered GO database mentioned above. The hits with probability >30 (out of 100) are kept. A score is calculated for each hit by:

15 $$Score=0.3\times \frac{prob}{100}+0.7\times \frac{matched\_residues}{query\_length}$$

in which $prob$ is the probability of hit, $matched\_residues$ is the number of residues matched with the hit, and $query\_length$ is the number of residues of the query protein. The constants $0.3$ and $0.7$ can generate the best performance based on evaluations (data not shown). A confidence score for each GO term of the hits is calculated similarity as in Eq. (14).

Besides searching against GO, we also use HHblits to search against Pfam^10^, KOG^15^, and COG^14^ with the same way of calculating scores and confidence. KOG^15^ is the eukaryote-specific version of COG^14^ that contains ortholog and paralog proteins.

GO terms predicted from protein domain architecture were integrated by executing tool multiPfam2go^13^. The Pfam domains detected by HHblits searching against Pfam are also input into multiPfamgo to get predicted GO terms.

All the GO terms are then pooled in the following way: the predictions from PSI-BLAST search against UniProtKB were all kept with original confidence scores; and the top *n* predictions from other tools were used. Different tool has a different value of *n* (1 to 5), which is determined by our evaluations that can generate the best performance. This step removes noises and help improves the performance. We assign a weight to each of the tools based on their area under the curve values such as in Fig. 2. We also tested other ways of pooling the GO terms, for example, weighted by their ${F}_{max}$ score based on evaluation. However, they did not achieve better overall performance. To ensure a fast speed for the web server, we do not include domain architecture or HHblits searching against PfamA. Removing these two tools saves computational time and does not affect performances based on our evaluations.

The pooled GO terms were then filtered by a Z-score of the confidence score:

16 $${Z}_{i}=\frac{Con{f}_{i}^{pool}-Av{g}^{pool}}{St{d}^{pool}}$$

in which $Con{f}_{i}^{pool}$ is the confidence score of term $i$, *Avg*^*pool*^ is the mean confidence score of all pooled GO terms, and $St{d}^{pool}$ is the standard deviation of the pooled confidence scores. The GO terms with Z >= 1 are kept. Our evaluation shows that this filtering step slightly increases the ${F}_{max}$ score.

The filtered GO terms (not prorogated) are clustered using affinity propagation^17^. The semantic similarities between GO terms are calculated based on Lin^13^ formula using the tool fastSemSim (https://sites.google.com/site/fastsemsim/). Because this tool is very slow, for the webserver of PANDA, we use a much faster tool with an improved algorithm named GOGO (http://dna.cs.miami.edu/GOGO/, manuscript submitted) to calculate semantic similarities between GO terms. The performance evaluations shown in this paper are based on Lin’s method. However, using GOGO will not largely change the performance as most of the low-quality GO terms have been eliminated by the first round of filtering.

The GO semantic similarities are directly used as the similarity measurement for clustering. In the affinity propagation clustering software, the preference of every GO term is set to a same value that is the average GO term similarity, which will reduce the number of clusters.

After clustering, an information content is calculated for each GO term as Eq. (11). The GO terms with higher depth value in the GO directed acyclic graph will have a higher information content, which means by using information content we preferably select GO terms with higher depth value. A score of each cluster is calculated as:

17 $$Scor{e}^{cluster}=\sum _{i=1}^{n}(Con{f}_{i}+I{C}_{i})$$

which is the sum of the confidence score and information content of every GO term in the cluster. A Z-score of each cluster’s $Scor{e}^{cluster}$ is calculated; and the clusters whose $Scor{e}^{cluster}$ are above a threshold Z-score (−2 was used) are kept. In this way, we remove the clusters that contain either low confident or less specific GO terms. All the remaining GO terms are used as final output together with their confidence available.

For PANDA server, if it finds exactly the same sequence (e-value equals zero and coverage is 100%) as the query, that hit is not used for prediction.

## References

[1] Ashburner M (2000). Gene ontology: tool for the unification of biology. Nature Genetics.

[2] Tian W, Skolnick J (2003). How well is enzyme function conserved as a function of pairwise sequence identity?. Journal of Molecular Biology.

[3] Falda M (2012). Argot2: a large scale function prediction tool relying on semantic similarity of weighted Gene Ontology terms. BMC Bioinformatics.

[4] Cozzetto D, Buchan DW, Bryson K, Jones DT (2013). Protein function prediction by massive integration of evolutionary analyses and multiple data sources. BMC Bioinformatics.

[5] Soding J, Biegert A, Lupas A (2005). The HHpred interactive server for protein homology detection and structure prediction. Nucleic Acids Research.

[6] Wang Z, Cao R, Cheng J (2013). Three-level prediction of protein function by combining profile-sequence search, profile-profile search, and domain co-occurrence networks. BMC Bioinformatics.

[7] Radivojac P (2013). A large-scale evaluation of computational protein function prediction. Nature methods.

[8] Wang Z, Eickholt J, Cheng J (2010). MULTICOM: a multi-level combination approach to protein structure prediction and its assessments in CASP8. Bioinformatics.

[9] Yang J (2015). The I-TASSER Suite: protein structure and function prediction. Nature methods.

[10] Bateman A (2004). The Pfam protein families database. Nucleic Acids Research.

[11] Li, W. *et al*. The EMBL-EBI bioinformatics web and programmatic tools framework. *Nucleic Acids Research*, gkv279 (2015). 10.1093/nar/gkv279 PMC4489272 25845596

[12] Wang Z (2011). A Protein Domain Co-Occurrence Network Approach for Predicting Protein Function and Inferring Species Phylogeny. PLoS ONE.

[13] Forslund K, Sonnhammer EL (2008). Predicting protein function from domain content. Bioinformatics.

[14] Tatusov RL (2003). The COG database: an updated version includes eukaryotes. BMC Bioinformatics.

[15] Koonin EV (2004). A comprehensive evolutionary classification of proteins encoded in complete eukaryotic genomes. Genome Biology.

[16] Powell, S. *et al*. eggNOGv4. 0: nested orthology inference across 3686 organisms. N*ucleic Acids Research*, gkt1253 (2013). 10.1093/nar/gkt1253 PMC3964997 24297252

[17] Frey BJ, Dueck D (2007). Clustering by passing messages between data points. Science.

[18] Huang Y, Niu B, Gao Y, Fu L, Li W (2010). CD-HIT Suite: a web server for clustering and comparing biological sequences. Bioinformatics.

[19] Jiang, Y. *et al*. An expanded evaluation of protein function prediction methods shows an improvement in accuracy. *Genome Biology* **17**, 10.1186/s13059-016-1037-6 (2016). 10.1186/s13059-016-1037-6 PMC5015320 27604469

[20] Clark WT, Radivojac P (2013). Information-theoretic evaluation of predicted ontological annotations. Bioinformatics.

[21] Cherry JM (1998). SGD: Saccharomyces genome database. Nucleic acids research.

[22] Altschul S (1997). Gapped BLAST and PSI-BLAST: a new generation of protein database search programs. Nucleic Acids Research.

[23] Remmert M, Biegert A, Hauser A, Söding J (2012). HHblits: lightning-fast iterative protein sequence searching by HMM-HMM alignment. Nature methods.

